# Impact of Group Asthma Education on Asthma Control and Emergency Room Visits in an Underserved New York Community

**DOI:** 10.1155/2019/5165189

**Published:** 2019-10-01

**Authors:** Asghar Ali, Sybil Goday Pena, Charnicia Huggins, Franklyn Lugo, Misbahuddin Khaja, Gilda Diaz-Fuentes

**Affiliations:** ^1^Division of Pulmonary and Critical Care Medicine, BronxCare Health System, 1650 Grand Concourse, Bronx, NY 10457, USA; ^2^Department of Pharmacy, BronxCare Health System, 1650 Grand Concourse, Bronx, NY 10457, USA; ^3^Department of Respiratory Care Services, BronxCare Health System, 1650 Grand Concourse, Bronx, NY 10457, USA

## Abstract

**Objective:**

Asthma education programs have been shown to be effective in decreasing health care utilization and improving disease control and management. However, there are few studies evaluating the outcomes of group asthma education. The aim of this study was to assess the impact of an outpatient adult group asthma education program in an inner-city-based hospital caring for an underserved population.

**Methods:**

We conducted a pre- and poststudy of all patients with asthma who participated in two structured group asthma education sessions led by a respiratory therapist, clinical pharmacist, and pulmonologist. The study period (January 2016 to April 2018) included the year before group education and the year after education. The primary outcomes were the number of patients requiring emergency room visits and hospital admission. The secondary outcomes included asthma control as assessed by Asthma Control Test scores, use of systemic corticosteroids, and change in test scores postintervention.

**Results:**

Eighty-eight patients received group education during the study period; 82 attended 2/2 sessions, and 6 attended 1/2 sessions. The study population was largely Hispanic (73%) or African American (25%) and had a mean age of 58 years. Most had moderate (57%) or severe (25%) persistent asthma. Significantly, fewer patients required emergency room visits in the postintervention period than in the preintervention period (20 visits vs. 42 visits, *p*=0.0002). Group education was also associated with increased asthma control (*p*=0.0043), decreased use of systemic corticosteroids (*p*=0.0005), and higher postintervention test scores (*p*=0.0001).

**Conclusions:**

Group asthma education provided by a multidisciplinary team in an inner-city hospital clinic caring for underserved and minority populations is feasible and may decrease utilization of health care resources when patients are educated and empowered to participate in their asthma management.

## 1. Introduction

Asthma is a common lung disorder characterized by bronchoconstriction and inflammation. According to the Centers for Disease Control and Prevention, 1 in 13 people have asthma, meaning that more than 26 million Americans, or approximately 8.3% of adults and children, have the disease [1].

Asthma represents a significant health and economic burden for patients, their families, and society. Between 2008 and 2013, the annual economic cost of asthma was more than $81.9 billion, including medical costs, and loss of work and school days [2].

The borough in New York City most affected by asthma is the Bronx; the prevalence of the disease in the population of the South Bronx is 10.7%, which is higher than that in New York City (10.2%) and nationwide (7.8%). According to community health profiles published by the New York City Department of Health and Mental Hygiene, the hospitalization rate for asthma in some South Bronx neighborhoods is three times that in the rest of New York City and 1.5 times that in the rest of the Bronx [3].

Rescue inhalers are the cornerstone of therapy for immediate relief of asthma exacerbations, while controllers are essential for patients with persistent asthma. Some of the barriers to asthma control include lack of education regarding correct inhaler technique, the wide range of inhaler devices available making the learning process more complex, and poor adherence to prescribed medications. The World Health Organization reports that adherence to medication among patients with chronic diseases is on an average only 50% in developed countries. This represents a tremendous challenge to population health efforts when success is determined primarily by adherence to long-term therapies [4].

The Global Initiative for Asthma (GINA) guidelines were developed to improve the care and control of the disease and to recommend asthma education for all affected individuals as an integral part of disease management [5].

Many studies have shown that asthma education improves outcomes, including asthma-related emergency room (ER) utilization and hospitalizations, unscheduled physician visits, days off work, and quality of life (QoL) [6].

Our institution, the BronxCare Health System, is located in the South Bronx and provides health care for a large underserved immigrant population with asthma. In a previous study, we showed that personalized, one-on-one asthma education had an impact on asthma control, ER visits, and hospital admissions [7]. However, providing asthma education in a group classroom format may have additional benefits that extend beyond individualized education. Benefits of group education in patients with diabetes have been reported, including increased effectiveness in comparison with individualized education [8]. Other researchers who reviewed studies of adults with chronic illness found that patients derived various benefits from group education, including improved self-management strategies and peer support [9].

The aim of this study was to evaluate the role of a group asthma education program in asthma control and health care utilization in our inner-city patient population.

## 2. Materials and Methods

This was a pre- and poststudy of all patients with asthma in our pulmonary clinic who participated in structured group education. The study period was from January 2016 to April 2018. Only those patients who were followed for one year before and one year after asthma education were included.

Outcomes were compared for the same group of patients during the preintervention and postintervention periods. The primary outcomes evaluated were the number of patients requiring ER visits and hospital admission for asthma exacerbation at our institution before and after intervention. The secondary outcomes were as follows: asthma control (assessed by the Asthma Control Test (ACT) score); requirement for systemic steroids (either prescribed at our institution or patient-reported); and change in asthma education test scores. All outcomes, except for the asthma education test score, were evaluated 12 months before and 12 months after asthma education.

### 2.1. Structure of the Asthma Education Program

Standardized group asthma education for 8–12 patients was offered in the pulmonary clinic by certified asthma educators, including bilingual (English and Spanish) respiratory therapist, clinical pharmacist, and pulmonologist. Education was provided in the patient's preferred language. Sessions were offered in English or Spanish.

The two education sessions consisted of meetings for two to two and a half consecutive hours per week. The standardized education tools used were based on the GINA guidelines and included a PowerPoint presentation, education booklets, a lung model, spacers, peak flow meters, and placebo inhalers for demonstration purposes. Both weekly sessions were held by the same educators.

The patients underwent a preeducation test of their knowledge on basic aspects of asthma and its management. This was followed by discussion and lectures on asthma. The teaching focused on the pathophysiology of asthma, its clinical presentation, triggers and trigger avoidance, environmental control, and management. Counseling regarding correct use of inhaler devices, peak flow meters, and spacers was included; patients were required to demonstrate appropriate use of inhalers and a peak flow meter. Regarding education on use of inhalers, the teach-back technique was used. Asthma educators developed and followed a standard check list which required demonstration of all steps twice with a placebo inhaler followed by patients' demonstration. On the second session, each patient demonstrated the inhaler technique to one of the educators.

No medications were distributed.

The patients learned and developed their own asthma action plan and completed their ACT.

The second session concluded with a postcourse test. All patients were provided with a healthy lunch/snack as well as a care package containing a peak flow meter, spacer, pen, and asthma-related literature. The attendees were given a personalized asthma action plan via a one-on-one teaching approach and had all their questions answered during this time. Both sessions included discussion on the impact of nutrition, healthy eating habits, and obesity and GERD on asthma.

The role of the pulmonologist during these sessions was of an educator, unless patients had specific clinical complaints like acute asthma exacerbation. Their medications were not changed nor they had a separate medical encounter with the pulmonologist.

A draft of agenda for each teaching session is shown in Table 1.

Patients attending the group education sessions were referred from our pulmonary clinic at the discretion of the pulmonologist. The practice at our clinic is that all patients been managed for obstructive airway disease undergo either spirometry or full pulmonary function tests as clinically indicated. Fractional exhaled nitric oxide (FeNO) is a quantitative, noninvasive, simple method of measuring airway inflammation that provides a complementary tool to other ways of assessing airway diseases in asthma patients. FeNO is available at our clinic, and it is performed as per the pulmonologist recommendation.

### 2.2. Severity of Asthma

To standardize the severity of asthma for the purpose of this study, we used established guidelines by the Global Initiative for Asthma (GINA) 2018 [5]. The guidelines categorize asthma severity as mild, moderate, or severe. Severity is assessed retrospectively from the level of treatment required to control symptoms and exacerbations, as follows:

- Mild asthma: well controlled with as-needed reliever medication alone or with low-intensity controller treatment such as low-dose inhaled corticosteroids (ICSs), leukotriene receptor antagonists, or low-dose theophylline.
- Moderate asthma: well controlled with low-dose ICS/long-acting beta2-adrenergic agonists (LABA) plus leukotriene receptor antagonists, and/or low-dose theophylline.
- Severe asthma: requires high-dose ICS/LABA plus leukotriene receptor antagonists, and/or low-dose theophylline or long-acting muscarinic antagonists (LAMA).

Asthma severity in our study was assessed by medication been used at the time of education.

### 2.3. Statistical Analysis

The analysis was performed on preeducation and posteducation data. A *t*-test was performed to determine if education had any impact on rates of adherence with asthma controller medication. The statistical analysis was performed using IBM SPSS software (version 19.0; IBM Corp., Armonk, NY, USA). The impact was considered statistically significant if the *p* value was ≤0.05.

The pre-education data served as the control group, and the postasthma education session data served as the intervention group.

The study was approved by the Institutional Review Board at our institution (approval number 09131809) and conducted in accordance with the amended Declaration of Helsinki.

## 3. Results

### 3.1. Demographic Data

A total of one-hundred and ten patients were invited to the group session. Eighty-eight patients (80%) received group education during the study period; 82 attended the 2-day group session, and 6 attended only one session.  Table 2 shows the characteristics of the patients at baseline.

The main reason for patients not to attend the education program or attend only one session was conflict with family responsibilities.

The patients had an average age of 58 years and showed a female predominance (75%). The majority (73%) of the group was Hispanic, and 25% were African American, reflecting the population served. Nine percent of patients were current smokers. The average body mass index was 31. The severity of persistent asthma was mild in 11% of cases, moderate in 53%, and severe in 35%. Comorbid conditions were found in up to 18% of the cohort, with obstructive sleep apnea and gastroesophageal reflux disease (GERD) being the most common.

In our cohort, 85 of the 88 (97%) patients had spirometry available, and FeNO was performed in 68 of the 88 (77%) patients.

We found a FeNO less than 25 ppb in 30 (44%), between 25 and 50 ppb in 18 (26%), and more than 50 ppb in 20 (29%) patients.

### 3.2. Primary Outcomes

During the posteducation period, there was a decrease in the number of patients requiring ER visits (preeducation (*n* = 42) and posteducation (*n* = 20); *p*=0.0002). There was also a decrease in the mean annual number of ER visits per patient from 0.86 before education to 0.44 after education (*p*=0.0066, 95%).

There was no significant between-group difference in the hospitalization rate or in the average number of hospital admissions per patient per year. A subgroup analysis of the 21 patients who required hospital admission showed that they needed more medications for asthma control and were likely to be receiving long-term systemic steroids, have more comorbid conditions, and have asthma/COPD overlap (Table 3).

### 3.3. Secondary Outcomes

The effect of group education on asthma control was evaluated using an ACT score ≥20 as per the GINA guidelines. Asthma education had a statistically significant (*p*=0.0043) effect on the number of patients with asthma and an ACT score ≥20 (28 (32%) before education versus 44 (50%) after education (*p*=0.0043)). We also found a significant decrease in the use of prescribed systemic corticosteroids from 51 patients before education to 28 patients after education (*p*=0.0005).

Knowledge and understanding of the basic concepts of asthma, its management, and anti-asthma medications as evaluated by the before and after education test scores were improved; the proportion of correct answers improved significantly from 72% to 86% (*p*=0.0001). A comparison of the outcomes is provided in Table 3.

### 3.4. Subgroup Analysis

Six (7.3%) patients attended only one of the education sessions; five were Hispanic and one was African American. One of those six patients was hospitalized for asthma, and he was Hispanic. Rest of the demographic and clinical characteristics were comparable to the remaining 82 patients. We found no differences in outcomes for patients who attended only one education session.

In addition, we compared the Hispanic group with the African American group. We had 64 Hispanic and 22 African American patients. We found no differences in demographic characteristics or comorbidities between the groups. Contrary to our expectations, severity of asthma between the groups was not statistically different; 81% of Hispanic patients had moderate or severe asthma compared with 82% of African American patients. There were more active cigarette smokers in the African American group (13.6%) than in the Hispanic group (7.8%), but this was not significant. Among the 21 asthmatic patients who required hospital admission, there were more Hispanics (71%) than African Americans (28.5%).

The subgroup analysis has a limited value due to the small number of patients.

## 4. Discussion

Our study shows that a comprehensive group asthma education program in an inner-city-based pulmonary clinic leads to decreased health care utilization. Reduced ER utilization as well as improved asthma control and decreased use of systemic steroids was achieved in our study participants. To our knowledge, this is one of the few studies that have used small group education sessions over a short period of time, i.e., 2 hours per week over 2 consecutive weeks. The purpose of the small group education was to improve asthma control and to utilize existing resources.

Data regarding the effect of group education in patients with asthma are sparse. Choy et al. investigated the efficacy of a hospital-based asthma education program in a low socioeconomic community in Hong Kong. In that study, 230 patients attended 2-hour asthma specialist respiratory nurse-led sessions that included education regarding pathophysiology of the disease, potential triggers, inhaler techniques, and self-management strategies, followed by a video session during a subsequent clinic visit. There were improvements in the inhaler technique and knowledge about asthma, as well as reductions in ER use and unexpected physician visits after education [10].

A Brazilian study compared the outcomes with an asthma education group (*n* = 26) and a control group (*n* = 27). Monthly education was provided for six months by a pulmonologist in training; this resulted in a decreased number of ER visits, better symptom control, and improved scores on the QoL questionnaire; however, there was no reduction in the number of hospital admissions [11]. The Brazilian study differed from ours in that it included different patients in the control and intervention groups, and education sessions comprised two sessions of one hour each.

Urek and colleagues compared the effects of three educational asthma control programs on asthma-related QoL in 60 adult patients with moderate persistent asthma. Each of the three groups contained 20 patients. The education programs compared were of individual verbal instruction (three one-hour sessions), written asthma information (asthma booklet), and group classes on asthma (four hours of group education). Researchers reported that asthma group classes and individual verbal education improved QoL and asthma control during three months of follow-up [12].

Similar to the study by de Oliveira and colleagues [11], we did not find a difference in hospital admissions after group education. We postulate that education may have failed in this subgroup of patients and acknowledge that they were likely to have more severe asthma attacks and more comorbidities. Identification of patients potentially requiring closer follow-up and additional education could be beneficial in terms of decreasing asthma-related admissions.

There are few studies comparing individual education versus group education on asthma. Prior study at our institutions with the same inner-city populations showed improvement in asthma control and decreased rate of ED utilization but no change in the hospitalization rate with individual education [7]. Three other studies reached the conclusion that both individual and group educations were beneficial and improved patient's outcomes. No study was able to conclude which format was better, yet there was a nonsignificant trend for long-term benefits in patients receiving group education. In addition, there was a wide variation in the delivery format [13–15].

Common comorbid conditions in our asthma cohort were obesity, obstructive sleep apnea, GERD, and asthma/COPD overlap. Obesity is a major risk factor for asthma in both children and adults. Obese patients are at increased risk of frequent and severe exacerbations and decreased QoL [11]. A study that compared 78 patients with asthma and 62 with COPD showed that patient education increased both health-related QoL and forced expiratory volume in one second in those with asthma but not in those with COPD [16].

Asthma and GERD are both common conditions and often coexist. The prevalence of symptoms of GERD has been found to be much higher in patients with asthma than in the general population. Almost 20% of our cohort reported having GERD, which could have contributed to their poor asthma control. However, treatment of GERD has not been shown to have an impact on asthma control or lung function [17]. Implementation of educational programs in health care has many barriers that are related to both patients and health care systems. Patient barriers include language and culture, lack of time, perception of low value of education in disease management, and financial limitations on attending education sessions; some of the system barriers include lack of ancillary support staff trained in asthma education, constraints on the amount of time pulmonologists have available for education, and issues related to payment by insurance carriers. These factors play a significant role when trying to educate communities in underserved areas like ours.

Strengths of our study are as follows: The first is the use of the same patients in the control and intervention groups, which allowed us to eliminate some of the potential environmental confounders and triggers that arise when comparing different groups of patients. The second strength is that we followed our patients for a relatively long period of time, i.e., one year. The third strength was an unforeseen and unmeasured consequence of group education, which was the development of a collaborative relationship not only between the patient and providers but also between the patients themselves. The education program allowed the patients to share personal experiences, to exchange contact numbers, and to call an educator for health advice instead of just going to the ER when they felt unwell. This was an important step for patients in that they could trust the health care system from which they were seeking attention. Finally, we attempted to compare two ethnic groups, Hispanics and African Americans; we did not find a significant difference between them. This could be due to the small numbers or ethnic differences.

The main limitations of the study are its pre-post retrospective design, which may have introduced a degree of selection bias. In general, patients in our community tend to seek care at the same hospital, but the possibility that some of the patients in our study visited other ERs or hospitals and their data were not captured cannot be excluded. Similarly, the results regarding use of systemic steroids were based only on data available in the electronic medical records and patient self-reports. Barriers for medication adherence were not evaluated either.

## 5. Conclusion

Our study supports the experience of other researchers regarding the effectiveness of asthma education programs in improving asthma management and education. Decreased use of systemic corticosteroids could potentially decrease the side effects of these agents. We have shown that implementation of group asthma education sessions in an inner-city-based hospital clinic caring for underserved and minority populations is feasible. It requires little institutional support and can be easily standardized and incorporated into asthma management. At our center, including a respiratory therapist and a clinical pharmacist into the clinic structure help us to meet other needs in patient care, such as performing pulmonary function tests, evaluation of medication, and management. Adherence to attendance at two sessions was very good. Due to few numbers of patients, we cannot conclude if one versus two group session is equally beneficial. Larger studies with well-designed programs and longer follow-up of patients are needed to evaluate the cost-effectiveness of educational programs with a different structure in various population settings. Furthermore, focusing on high-risk patients and patients who visit the ER or patients who are admitted to hospital on a regular basis could improve care even further. Long-term follow-up of patients with asthma who receive education is paramount for determining if and when reinforcement sessions are needed. Group programs are simpler to administer and potentially patients could be motivated and be confident of their ability to control their conditions. They have the opportunity to share their experiences with other patients as well.

## Figures and Tables

**Table 1:** Session agenda.

Time	Session 1	Session 2
	Discussions and teaching goals	
60 min	Introduction and educational objectives of the program Precourse test	Recap salient points from session 1
	Short, PowerPoint presentations/videos discussing (i) Pathophysiology of asthma (ii) Triggers and allergens (iii) Techniques for trigger control including infection control (iv) Tobacco exposure and asthma	Hand on discussion of (i) Action plan (ii) Use of inhalers and peak flow meters

10 min	Break	

10–15 min	Lunch and questions—participation of all educators	

15 min	PowerPoint presentations/video clips discussing (i) Basic pharmacology of asthma medications (ii) Rescue versus controllers	Group discussion regarding asthma and diet (led by either a nutritionist or a pulmonologist)

45 min	Hand on teaching and patient demonstration of (i) Inhalers and how to use them (ii) Peak flow meters and spacers (iii) Maintenance of asthma equipment (iv) Group questions addressed—participation of all educators	Hand on teaching and patient demonstration of (i) Personal action plan for each patient (ii) Peak flow meters and spacers (iii) Use of inhalers (iv) Group questions addressed (v) Posttest and review questions with the group participation of all educators

10 min		Patient written feedback of the program distribution of completion diploma group pictures

**Table 2:** Demographic and clinical characteristics.

Characteristic	*n* = 88	*n* = 21 asthma admissions
Age, years	58.89 ± 11.08	60 ± 8.18
Sex		
Male	22 (25%)	3 (14.2%)
Female	66 (75%)	18 (85.7%)
Race		
Hispanic	64 (72.7%)	15 (71.4%)
African American	22 (25%)	6 (28.6%)
Others	2 (2.27%)	0
Tobacco, active smoker	8 (9.09%)	4 (19.0%)
Body mass index	31.60 ± 7.08	33.81 ± 7.34
Spirometry available	85 (97%)	
FeNO	68 (77%)	
Severity of asthma based on GINA 2018		
Mild (step 1 or 2)	16 (18.2%)	3 (14%)
Moderate (step 3)	50 (56.8%)	12 (57.1%)
Severe (step 4-5)	22 (25%)	6 (28.6%)
Number of controller medications for asthma		
ICS alone	19 (21.6%)	3 (14.2%)
ICS + LABA	34 (38.6%)	7 (33.3%)
ICS + LABA + LAMA	31 (35.2%)	11 (52.4%)
Leukotriene inhibitors	64 (72.7%)	21 (100%)
Theophylline/roflumilast	8 (9.1%)	2 (9.5%)
Long-term systemic steroids	5 (5.8%)	3 (14.2%)
Comorbidities		
Obstructive sleep apnea	16 (18.2%)	5 (23.8)
Gastroesophageal reflux	15 (17.0%)	5 (23.8)
Congestive heart failure	5 (5.7%)	0
Asthma/COPD overlap syndrome	7 (7.95%)	4 (19.0%)
Bronchiectasis	2 (2.3%)	1 (4.7%)

COPD, chronic obstructive pulmonary disease; ICS, inhaled corticosteroids; LABA, long-acting beta2-adrenergic agonists; LAMA, long-acting muscarinic agents.

**Table 3:** Outcomes after asthma education.

Outcome	Preeducation (*n* = 88)	Posteducation (*n* = 88)	*p* value
Patients requiring ER visits, *n*	42	20	0.0002
Average number of ER visits/patient/year	0.86	0.44	0.0066
Patients requiring hospitalization, *n*	21	19	0.1812
Average number of hospitalizations/patient/year	0.39	0.26	0.2236
Patients with controlled asthma (ACT ≥20), *n*	28	44	0.0043
Mean test score (%)	72.27	85.92	0.0001
Use of systemic steroids (number of patients)	51	28	0.0005

ACT, Asthma Control Test; ER, emergency room.

## Data Availability

This is a retrospective, computerized database (CPRS) study performed within the BronxCare Health System. The information used in this research project was recorded in a way that patients cannot be identified. Data used to support the findings of this study are available from the corresponding author upon request.

## Abbreviations

ACT: Asthma Control Test
COPD: Chronic obstructive pulmonary disease
ER: Emergency room
GERD: Gastroesophageal reflux disease
GINA: Global Initiative for Asthma
QoL: Quality of life
FeNO: Fractional exhaled nitric oxide.
